# Analysis of infectious spondylodiscitis: 7-years data

**DOI:** 10.12669/pjms.346.15717

**Published:** 2018

**Authors:** Gulay Okay, Yasemin Akkoyunlu, Sibel Bolukcu, Bulent Durdu, Ismail Necati Hakyemez, Meliha Meric Koc

**Affiliations:** 1 *Gulay Okay, MD. Department of Infectious Diseases and Clinical Microbiology, Bezmialem Vakif University, Istanbul, Turkey*; 2 *Yasemin Akkoyunlu, Associate Professor. Department of Infectious Diseases and Clinical Microbiology, Bezmialem Vakif University, Istanbul, Turkey*; 3 *Sibel Bolukcu, MD Department of Infectious Diseases and Clinical Microbiology, Bezmialem Vakif University, Istanbul, Turkey*; 4 *Bulent Durdu, Assistant Professor. Department of Infectious Diseases and Clinical Microbiology, Bezmialem Vakif University, Istanbul, Turkey*; 5 *Ismail Necati Hakyemez, Associate Professor. Department of Infectious Diseases and Clinical Microbiology, Bezmialem Vakif University, Istanbul, Turkey*; 6 *Prof. Meliha Meric Koc, Department of Infectious Diseases and Clinical Microbiology, Bezmialem Vakif University, Istanbul, Turkey*

**Keywords:** Infectious spondylodiscitis, Pyogenic, Tubercular, Brucellar, Clinical features, Diagnosis

## Abstract

**Objective:**

Infectious spondylodiscitis (SD) is an infectious disease that is rare and difficult to diagnose due to its non-specific clinical features. In this study, we aimed to describe the clinical and diagnostic features of infectious spondylodiscitis.

**Methods:**

All patients who were diagnosed with SD at our hospital during a 7-year period from January 1, 2011 through December 31, 2017 were included in the study. Spondylodiscitis is divided into the following three types: pyogenic, tuberculous, and brucellar. Clinical and laboratory data were collected retrospectively from the medical records of the patients.

**Results:**

Of the 118 patients, 66 (55.9%) were female, 81 (68.6%) had pyogenic SD (PSD), 21 (17.8%) had tuberculous SD (TSD), and 16 (13.6%) had brucellar SD (BSD). The mean age was 59.3 ± 14.6 years. Leucocytosis was significantly higher in patients with PSD (p=0.01) than in patients with other types of SD. Thoracic involvement (47.6%) was significantly higher in patients with TSD (p=0.005) than in other patients. Sacral involvement (12.5%) was significantly higher in patients with BSD (p=0.01) than in other patients. Paravertebral abscess formation (42.8%) occurred most frequently in patients with TSD. Microbiologic agents were defined in 50% (18/36) of the surgical specimens and in 12.5% of the fine needle aspiration biopsy (FNAB) specimens. Staphylococcus aureus was the most common microbiological agent in patients with PSD. Spinal surgery was defined as a risk factor for PSD (p = 0.0001). Binary logistic regression analysis revealed that female gender, thoracic involvement and night sweats were the predictive markers for TSD (OR 4.5 [95% CI 1.3-15.3] and OR 5 [95% CI 1.7-14.6]).

**Conclusion:**

PSD is the most frequent form of SD. Leucocytosis is most common in patients with PSD. Thoracic involvement and paraspinal abscess were prominent in patients with TSD. Sacral involvement was most common in patients with BSD. Thoracic involvement, female gender and night sweats were the predictive markers for TSD. The microbiological culture positivity rate was higher in surgical specimens compared to FNAB specimens. The need for surgical treatment was most common in patients with TSD.

## INTRODUCTION

Infectious spondylodiscitis (SD) is defined as an infectious disease affecting the vertebral body, the intervertebral disc, and/or adjacent paraspinal tissue.^1^ Although rare, SD is the main manifestation of haematogenous osteomyelitis in patients older than 50 years of age.^2^ Its incidence seems to be increasing as a result of the increase in spinal instrumentation and surgery and with a higher life expectancy of older patients with chronic debilitating diseases.^3^,^4^

The clinical diagnosis is still a challenge. In adults, the symptoms and clinical signs of patients may be nonspecific, and early diagnosis may be difficult. Back pain is the most frequent initial symptom of SD, followed by fever. Neurological deficits are less common.^3^,^5^,^6^ Predisposing factors for SD are advancing age, diabetes mellitus, intravenous drug abuse, HIV, malignancy, chronic steroid usage, renal failure, septicaemia, recent spinal surgery and intravascular devices.^3^,^5^,^7^

The most frequent pathogen is *Staphylococcus aureus*, followed by *Escherichia coli* in pyogenic spondylodiscitis. Gram-negative aerobic bacteria and *Candida* spp.-related infections are seen mostly in intravenous drug abusers, immunosuppressed individuals, or postoperative patients. *Mycobacterium tuberculosis* and *Brucella* spp. are less often involved in SD.^8^,^9^ The location of the infection is mainly in the lumbar spine, followed by the thoracic and the cervical spine.^5^,^8^-^10^

The aim of this study was to describe and to evaluate the bacteriological agents, clinical features, and laboratory and radiological findings in pyogenic, brucellar, and tuberculous spondylodiscitis patients for differential diagnosis and management.

## METHODS

This study was conducted at the BezmialemVakif University, Medical School Hospital (550 beds) in Istanbul. All patients with SD who were admitted to our hospital during a 7-year period from January 1, 2011 through December 31, 2017 were included.

Spondylodiscitis is divided into the following three types: pyogenic SD (PSD), tuberculous SD (TSD), and brucellar SD (BSD). The diagnosis of SD is made clinically, radiologically, and microbiologically. There are clinical signs (pain and/or fever), elevated inflammatory markers (leucocytes, C-reactive protein (CRP), erythrocyte sedimentation rate (ESR)), radiologic abnormalities (magnetic resonance imaging (MRI), computed tomography (CT)) and/or positive culture (blood and/or CT-guided fine needle aspiration biopsy (FNAB) and/or surgical specimen) and/or histopathologic examination.

The microbiological diagnosis was determined as follows: Surgical and biopsy samples were sent from the clinics to the microbiology laboratory and inoculated using blood agar (Becton–Dickinson, USA), eosin methylene blue agar (Becton–Dickinson, USA), and chocolate agar (Becton, Dickinson, USA) and incubated aerobically for 24-48 hours in a CO_2_ incubator at a temperature range of 35 ± 2 °C. Haemoculture tubes (Becton–Dickinson, USA) sent to the microbiology laboratory were placed into the BACTEC FX (Becton–Dickinson, USA) device and programmed for an incubation period of five days. In the samples with positive signals during this period, first Gram staining was performed from the bottle, after which incubations were conducted in blood agar (Salubris, Turkey), EMB agar (Salubris, Turkey) and chocolate agar (Salubris, Turkey) at 37 °C for 18-24 h. The bacteria that grew were defined using conventional methods and automated systems (VITEK® 2 Compact (BioMérieux, France), and antimicrobial susceptibility results obtained using the VITEK-2 were evaluated according to the Clinical and Laboratory Standards Institute (CLSI) (2013) criteria. The strains identified using the Vitec® 2 Compact (BioMérieux, France) device were also examined using the Vitec® MS (MALDI-TOF) (BioMérieux, France) device for identification and confirmation.

The radiological diagnosis of SD was established according to the following findings: Magnetic resonance imaging (MRI) showed hypointense discs and vertebral bodies in T1-weighted images and hyperintense signals of the same structures in T2-weighted images, as well as thickening of the paravertebral soft tissue and/or involvement within the vertebral canals; Computed tomography (CT) revealed areas of osteolysis, bone erosion, or vertebral endplate geodes.^1^,^3^,^5^,^11^

The diagnosis of TSD was based on the bacterial yield in specimens (CT-guided FNAB and/or surgical specimen) and/or acid-fast bacilli positivity in Ziehl-Neelsen stained slides or caseous granulomatous inflammation in histopathological samples. İf the culture was not positive, the diagnosis was made using clinical, laboratory and radiological and/or histopathological findings combined with a history of tuberculosis. The diagnosis of BSD was made by the clinical findings of brucellosis and the detection of *Brucella spp*. in the haemoculture or a positive Brucella agglutination test at a titer ≥ 1/160.

The diagnosis of PSD was made by clinical and laboratory findings and culture positivity and/or ruling out BSD and TSD. Fungal SD was included in the PSD group. The PSD cases that developed after spinal surgery were described as nosocomial PSD. Clinical and laboratory data were collected retrospectively from the medical records of the patients.

Data were analysed using SPSS, version 16.0 (SPSS). Data were expressed as the means ± standard deviation and the medians (IQR). In the comparison of categorical data, the chi-square or Fisher’s exact probability test was used. The Student’s t-test and Mann-Whitney U test were used for the binary comparison of the groups’ averages. The laboratory data among the three groups were compared using analysis of variance (ANOVA), and Tukey’s and Tamhane’s post hoc tests were used. The Kruskal-Wallis test was used for non-parametric data. Binary logistic regression (“forward: LR” method) was used for multivariate analysis. A p-value < 0.05 was considered significant.

### Ethic committee approval

This retrospective study was approved by the regional ethical authority (Ethic committee of Bezmialem Vakif University. The registration date and number is 08/05/2018 and 11/105).

## RESULTS

A total of 118 patients with SD were included in the study. A total of 66 (55.9%) patients were female, and the mean age was 59.3±14.6 years (range: 19-86 years). There was no significant difference in age between the groups (p=0.32). TSD was significantly higher in females (p=0.03) (Table-I). most frequent clinical symptoms are shown in Table-II. Night sweats were more common in patients with BSD (25%) and TSD (19%) than in those with PSD (1.2%). In the univariate analysis, night sweats were significantly higher in patients with BSD (p=0.0004). A history of spinal surgery, diabetes mellitus, chronic renal disease and malignancy were present in 30.5, 15.3, 9.3, and 8.5% of patients in our study, respectively. Spinal surgery was a risk factor for PSD (p=0.0001).

**Table-I:** Laboratory findings and frequency distribution of infectious spondylodiscitis by age and gender.

		Pyogenic 81 (68.6)	Tuberculous 21 (17.8)	Brucellar 16 (13.6)	Total 118 (100)	p
Age (mean±SD)		60.7±14.4	55.7±15.7	56.8±13.5	59.3±14.6	0.32^a^
Gender n (%)	Female	40 (49.4)	17 (80.9)*	9 (56.2)	66 (55.9)	0.03^b^
	Male	41 (50.6)	4 (19.1)	7 (43.7)	52 (44.1)
CRP median (IQR)		4(1.1-9.3)	3(1.7-7)	4(1.7-8.7)	3.9(1.3-8.3)	0.72^c^
ESR (mean±SD)		55.7±32.3	64±22.4	54.6±23.8	57±29.7	0.36^a^
Leucocytosis n(%)		25(30.9)**	3(14.3)	0	28(23.7)	0.01^d^

a ANOVA test,

b chi-square test,

c Kruskal-Wallis test,

d Fisher’s exact probability test;

* Tuberculous spondylodiscitis was significantly higher in women;

** Leucocytosis was significantly higher in patients with pyogenic spondylodiscitis; CRP: C-reactive protein, ESR: erythrocyte sedimentation rate

**Table-II:** Clinical manifestations in patients with infectious spondylodiscitis

Signs and symptoms	Pyogenic Spondylodiscitis n=81 (%)	Brucellar Spondylodiscitis n=16 (%)	Tuberculous Spondylodiscitis n=21 (%)	Total n=118 (%)	p
Pain	79 (97.5)	15 (93.7)	21 (100)	115 (97.5)	0.42^a^
Fever	9 (11)	4 (25)	4 (19)	17 (14.4)	0.25^a^
Night sweats	1 (1.2)	4 (25)*	4 (19)	9 (7.6)	0.0004^a^
Weight loss	3 (3.7)	2 (12.5)	2 (9.5)	7 (5.9)	0.15^a^

a Fisher’s exact probability test;

* The incidence of night sweats was significantly higher in patients with brucellar spondylodiscitis.

Leucocytosis was detected in 28 (23.7%) cases and significantly higher in patients with PSD (p=0.01). The mean ESR was 57 ± 29.7 mm/h, and the CRP median value was 3.9 mg/dL (IQR, 1.3-8.3). There were no differences in ESR and CRP averages between the groups (p=0.36; p=0.72) (Table-I).

For radiological examination, MRI was performed in 115 cases, and CT imaging was performed in three cases. The distribution of affected vertebral regions and complications by types of SD are shown in Table-III. Lumbar involvement (50.6%) was more common in patients with PSD compared to patients in the other groups but was not statistically significant (p=0.18). In the PSD group, lumbosacral involvement was the second most common area of the spine affected. Thoracic involvement (47.6%) was significantly higher in the TSD group (p=0.005). Lumbar involvement (33.3%) was the second most common area of the spine affected in the TSD group. In the BSD group, lumbar involvement was more common. Sacral involvement was significantly higher in patients with BSD (p=0.01). Complications (arachnoiditis, paravertebral and psoas abscesses) were detected in 47 (39.8%) patients. Complications were detected in 37% (30/81) of patients with PSD, 52.3% (11/21) of patients with TSD, and 37.4% (6/16) of patients with BSD (Table-III).

**Table-III:** Imaging findings in patients with pyogenic, brucellar and tuberculous spondylodiscitis.

	Pyogenic Spondylodiscitis n=81(%)	Brucellar Spondylodiscitis n=16(%)	Tuberculous Spondylodiscitis n=21(%)	Total n=118(%)	p
Affected level					
Cervical	4 (4.9)	1 (6.2)	0	5 (4.3)	0.63^a^
Thoracic	12 (14.8)	4 (25)	10 (47.6)*	26 (22.1)	0.005^a^
Lumbar	41 (50.6)	5 (31.2)	7 (33.3)	53 (44.9)	0.18^b^
Lumbosacral	22 (27.2)	2 (12.5)	2 (9.5)	26 (22.1)	0.19^a^
Sacral	0	2 (12.5)**	0	2 (1.7)	0.01^a^
Thoracolumbar	2 (2.5)	2(12.5)	2 (9.5)	6 (5.1)	0.09^a^
Complications	30 (37)	6 (37.4)	11 (52.3)	47 (39.8)	0.46^a^
Paravertebral abscesses	19 (25)	5 (31.2)	9 (42.8)	33 (27.9)	0.17^a^
Psoas abscesses	5 (6.6)	1 (6.2)	2 (9.5)	8 (6.7)	0.85^a^
Arachnoiditis	6 (7.9)	0	0	6 (5.1)	0.43^a^

a Fisher’s exact probability test,

b chi-square test;

* The incidence of thoracic involvement**** was significantly higher in patients with tuberculous spondylodiscitis;

** The incidence of sacral involvement**** was significantly higher in patients with brucellar spondylodiscitis.

Fine-needle aspiration biopsy was performed in 27% (32/118) of patients using the CT-guided method. Samples were collected during surgery in 30.5% (36/118) of the patients. Microbiological agents were detected in 18 surgical specimens, four CT-guided FNABs and 10 haemocultures. Tuberculous bacilli were detected in four samples (one surgical specimen and three CT-guided FNABs). Brucella bacilli were detected in one haemoculture. In one patient with PSD, the same agent was detected in both the haemoculture and surgical specimens. Microbiological agents were detected in 50% (18/36) of the surgical specimens and 12.5% (4/32) of CT-guided FNABs.

Of the patients with PSD, 26 (32%) patients had a microbiological diagnosis. Among them, the most frequent agent was *S. aureus* (15/26), and three of them were methicillin-resistant. The distribution of microbiological agents is shown in Table-IV. The condition was acquired nosocomially in 45 PSD patients. The most common agent in the nosocomial pyogenic group (n=15) was methicillin-sensitive *S. aureus* (n=4). Other microorganisms detected in the cases of the nosocomial pyogenic group were two cases of methicillin-resistant *S. aureus*, two *Acinetobacter* spp., two *E. coli*, one *Pseudomonas aeruginosa*, two *Enterobacter* spp., and two cases of yeast. Of the PSD group, 28 (34.5%) patients underwent spinal surgery for treatment. In the nosocomial PSD group, the need for surgery as treatment was significantly high (n=20, p=0.03). In patients with PSD, antibiotic treatment was prescribed according to the antibiotic susceptibility results in addition to the surgical treatment.

**Table-IV:** Microorganisms isolated from patients with pyogenic spondylodiscitis.

Microorganisms	n (%)
***Gram-positive cocci***	
*Staphylococcus aureus*	15 (18.5)
*Enterococcus spp.*	1(1.2)
***Gram-negative bacilli***	
*Acinetobacter spp.*	2 (2.5)
*Escherichia coli*	2 (2.5)
*Pseudomonas aeruginosa*	2 (2.5)
*Enterobacter spp.*	2 (2.5)
Yeast	2 (2.5)
*Candida albicans*	
*Acremonium spp.*	

In patients with TSD, four had culture positivity, and nine had pathological findings specific for tuberculosis (granulomatous and caseous inflammation), i.e., a history of latent tuberculosis, and/or a positive purified protein derivative (PPD) test, and/or a specific finding on MRI. Eight (38%) of the patients with TSD underwent surgical treatment. All the patients with BSD were positive for the Brucella agglutination test. In one case of BSD, the haemoculture was positive. Two (12.5%) of the patients with BSD underwent surgical treatment. In patients with TSD, antituberculosis drugs were administered, and a combination of rifampicin and doxycycline were used in patients with BSD.

The mean duration of treatment in patients with TSD, BSD, and PSD was 434.2±150.5 days, 337.8±57.8 days, and 148.6±120.9 days, respectively. The most common treatment choice for the pyogenic group was β-lactam antibiotics (39.5%), which had a significantly high rate of use in the community acquired PSD group (p=0.003).

Binary logistic regression analysis was performed for the three groups using the same variables. Some findings were found to be predictive markers only in the TSD group. Binary logistic regression analysis revealed that female gender, thoracic involvement and night sweats were the predictive markers for TSD (OR 4.5 [95% CI, 1.3-15.3] and OR 5 [95% CI, 1.7-14.6], respectively) (Table-V).

**Table-V:** Binary logistic regression analyses of risk factors for tuberculous spondylodiscitis.

	OR	95% CI OR	p
Gender (Female)	4.4	1.29-15.08	0.02
Thoracic involvement	6.1	2-19.1	0.002
Night sweats	6.3	1.27-31.8	0.01

(Independent risk factors: Gender, thoracic involvement, thoracolumbar involvement, cervical involvement, sacral involvement, lumbar involvement, lumbosacral involvement, weight loss, night sweats, fever, pain, diabetes mellitus, leucocytosis)

## DISCUSSION

Infectious spondylodiscitis is an uncommon, mainly haematogenous disease that generally affects elderly patients.^9^,^12^ Diagnosis can be difficult because the disease is rare, and the signs and symptoms are nonspecific. According to the literature^9^,^13^, spondylodiscitis mostly affects males. However, in some previous studies^14^,^15^, the incidence in females was slightly higher compared to that in males, which is similar to the finding in the current study. In our study, TSD was significantly higher in females (p=0.03). In another study of SD^13^, the number of females affected was higher than the number of males in the TSD group. Infectious spondylodiscitis occurs in all age groups, but the incidence is high especially in advanced age groups. Consistent with the literature^9^,^12^, in our study, the mean age of patients was 59.3±14.6 years.

Although brucellosis and tuberculosis are endemic in our country, most of the SD patients had PSD (68.6%), which is similar to the results reported in the literature.^13^,^16^ This may be due to the high number of nosocomial cases of PSD in our patients. TSD and BSD accounted for 17.8% and 13.6% of the study group, respectively. Some studies have reported different results. In one study, TSD was the most common type reported (43%), followed by PSD and BSD^15^, and in another study, BSD was the most commonly reported condition (42.7%).^17^

Comorbidities such as diabetes mellitus, chronic renal disease and malignancy are associated with the development of SD.^8^ Patients with chronic renal failure are at a higher risk of tuberculosis compared to the general population.^18^ In our study, diabetes mellitus (15.3%) and chronic renal disease (9.3%) were the most common comorbidities.

Back and cervical pain are the most common symptom of SD.^2^,^19^ Various studies have reported fever in 12-54% of patients.^17^,^20^ In our study, the most common symptom was back pain regardless of the aetiology (97.5%), and fever was observed in only 14.4% of all patients. Of the patients in our series, 17 (7.6%) patients had night sweats, and nine (5.9%) patients experienced weight loss. Fever and back pain were not significantly different among the three groups; however, night sweats were more common (25%) reported among the patients with BSD (p=0.0004), which was similar to the findings in a previous study.^14^ The logistic regression analysis revealed that night sweats were a predictive marker for TSD. In another study, the incidence of night sweats was similar in patients with BSD and TSD.^21^

Among the inflammatory markers, the leucocyte count, ESR and CRP are helpful in the evaluation of patients, but specificity is lacking.^2^ In the current study, leucocytosis was significantly higher in patients with PSD than in those with TSD and BSD (p=0.01), which was similar to the results of other studies^13^,^14^ We found that there was no difference in the means of ESR and CRP among the three groups (p=0.36; p=0.72, respectively). Some studies found that ESR and CRP were significantly higher in the pyogenic group than in the other group.^14^ In another study, ESR was significantly higher in the tuberculous group compared to that in the other groups.^17^

Because of its sensitivity and specificity, MRI is the examination of choice for diagnosing spondylodiscitis.^11^ In our study, MRI was used in most of the patients (115/118), and CT was used only in three patients due to the presence of metal instruments (MRI could not be performed). Infectious spondylodiscitis most commonly affects the lumbar vertebra, followed by the thoracic, cervical and sacral regions.^13^,^22^,^23^ In our patients, the most affected region was the lumbar (44.9%) area followed by the lumbosacral (22.1%) region, which is similar to that reported in the literature. Lumbar involvement in the pyogenic group was highest but not statistically significant (p=0.18). Thoracic involvement was significantly highest in the TSD group of our series (p=0.05), which was similar to that in the literature^24^,^25^, and in logistic regression analysis, it is a predictive marker for TSD. Sacral involvement was significantly higher (p=0.01) in the BSD group than in the other groups, which is similar to the results of a previous study.^21^ In our series, abscess formation (paravertebral and psoas) was detected in all three groups and the incidence was highest in patients with TSD (52.4%) but not statistically significant (p=0.17, *p*=0.85, respectively). In a study involving patients with TSD, the most common finding at baseline was abscess formation (69%).^24^ Another study reported that a significantly higher rate of TSD patients had a psoas abscess.^17^

It is difficult to determine the microbiological aetiology of SD. Surgical material and CT-guided FNAB have been reported to help in the determination of the microbiological aetiology at various rates.^12^,^14^,^17^ A culture of a biopsy specimen, whether the specimen is obtained with the use of a CT-guided method or an open technique, has a higher overall diagnostic yield than does a blood culture.^5^ In a study involving 19 PSD patients, the aetiology was identified in 52.6% of the patients by CT-guided FNAB.^17^ In our study, the microbiological agent was identified in 50% of surgical specimens and in only 12.5% by CT-guided FNAB. In our patients, the low percentage of culture positivity using CT-guided FNAB can be attributed to previous antibiotic use or failure to collect samples from the appropriate region.

In the current study, 68.6% of patients with SD had PSD, and 38.1% of these cases were acquired nosocomially. The most commonly affected vertebrae were located in the lumbar region, which is similar to that reported in the literature.^3^ The aetiological agent was yielded in 32% of patients with PSD. As in many previous studies, the most common aetiological agent in PSD was *S. aureus* followed by Gram-negative bacteria.^16^,^20^ In previous studies, Gram-negative bacilli were reported as causative agents in 4.2%-27% of patients with PSD^7^,^12^,^20^ In our patients with PSD, Gram-negative bacilli were identified in 10% of patients, and most of them (87.5%) were in the nosocomial group, which is similar to that reported in the literature.^7^,^12^,^20^ The majority of the microorganisms that caused nosocomial PSD were resistant strains such as *Acinetobacter* spp., *P. aeruginosa*, *Enterobacter* spp. and methicillin-resistant *S. aureus*

There are no data from controlled studies that suggest the optimal duration of therapy; however, appropriate antibiotics should be applied intravenously for 2–4 weeks or until CRP levels decrease.^1^,^5^ Oral antibiotic treatment is continued for a total of 6 to 12 weeks.^1^,^5^ A recent prospective randomized study showed no difference between a 6-week treatment and a 12-week treatment.^26^ In our study, the mean duration of treatment in TSD, BSD, and PSD was 434.2±150.5 days; 337.8±57.8 days; and 148.6±120.9 days, respectively. According to the results of the antibiotic susceptibility test, glycopeptides, daptomycin, linezolid, fluoroquinolones, or a β-lactam antibiotic were administered to patients with PSD, which is similar to that reported in the literature.^2^,^7^ In our study, the most common antibiotic of choice to treat PSD was a β-lactam agent (39.5%). In our study, surgical treatment was needed in addition to medical treatment in 34.5% of patients with PSD. Surgical treatment was performed for the following reasons: abscess drainage, debridement, implant removal, and vertebrae stabilization.

Brucellar spondylodiscitis is endemic in many parts of the world, especially in Mediterranean countries. A study from the Mediterranean Region showed that 33.4% of all spondylodiscitis cases were due to brucellosis.^16^ Two other studies from this region showed an incidence of BSD of 24% and 21%, respectively.^13^,^15^ In our study, BSD accounts for 14% of SD patients, which is slightly lower than that reported in recent studies, although Brucella is endemic in our country. All of the BSD patients were diagnosed using a Brucella agglutination test, and in one patient, the haemoculture was positive. In most of our patients with BSD, lumbar and lumbosacral involvement was present, and sacral involvement was significantly more common than in the other patients (p=0.01), which is similar to that reported in the literature.^21^

In endemic countries, tuberculosis is important in the differential diagnosis of SD patients, and the incidence of TSD in patients was high. In previous studies from endemic countries, TSD accounted for 17-43% of SD cases.^13^-^15^,^17^ The rate of TSD in patients was 17.8% in our study as a result of the endemic nature of tuberculosis in our country. Thoracic vertebrae involvement was significantly more common in patients with TSD (47.6%, p=0.05) in our series, which is similar to that reported in previous studies.^14^,^17^ In some studies, lumbar involvement was more frequently observed in TSD patients.^13^ Using logistic regression analysis, we detected thoracic involvement, female gender and night sweats as significant predictive markers for TSD. Paravertebral and psoas abscesses were present in 52.3% of patients with TSD; therefore, 38% of them needed surgical treatment in addition to medical treatment. For the treatment of TSD, four antituberculous drugs were administered for the first two months of therapy, followed by two drugs for 10 months. All the patients with TSD were administered antituberculous therapy for one year, and if necessary, this period was extended for several months depending on the clinical and laboratory findings. In the majority of our tuberculosis cases, we did not detect any immunosuppressed condition. Only two patients had chronic renal failure, and two patients had malignancy.

## CONCLUSION

SD is seen in advanced age and is more common in women. Although tuberculosis and brucellosis are endemic in our country, PSD is more common than TSD and BSD. Most of the patients had back pain. Leucocytosis is most common in patients with PSD. Surgical specimens are more useful than samples procured using CT-guided FNAB for the detection of the microbiological agent. Sacral involvement was most common in patients with BSD, and thoracic involvement was most common in patients with TSD. Thoracic involvement, female gender and night sweats were predictive markers for TSD. Abscess formation and the need for surgical treatment were most common in patients with TSD.
